# An Update of Transition Metal-Catalyzed Decarboxylative Transformations of Cyclic Carbonates and Carbamates

**DOI:** 10.3390/molecules24213930

**Published:** 2019-10-31

**Authors:** Linhong Zuo, Teng Liu, Xiaowei Chang, Wusheng Guo

**Affiliations:** Frontier Institute of Science and Technology (FIST), Xi’an Jiaotong University, Xi’an 710045, Chinaliuteng1992@stu.xjtu.edu.cn (T.L.); changxw16@163.com (X.C.)

**Keywords:** carbonates, carbamates, decarboxylation, annulation, transition metal catalysis, heterocycles

## Abstract

Functionalized cyclic organic carbonates and carbamates are frequently used in a number of transition metal-catalyzed decarboxylative reactions for the construction of interesting molecules. These decarboxylative transformations have attracted more and more research attention in recent years mainly due to their advantages of less waste generation and versatile reactivities. On the basis of previous reviews on this hot topic, the present review will focus on the development of transition metal-catalyzed decarboxylative transformations of functionalized cyclic carbonates and carbamates in the last two years.

## 1. Introduction

Decarboxylation is generally associated with steps of C–C bond cleavage and CO_2_ generation. As the sole byproduct of decarboxylative reactions, CO_2_ is non-flammable and non-toxic and can be easily removed from the reaction system; thus, decarboxylative chemistry has become a very important and attractive strategy in organic synthesis. Carboxylate groups have served as miscellaneous connection points in the construction of various organic compounds [1,2,3,4,5,6]. In contrast, cyclic organic carbonates and carbamates enabled new reactivities that go beyond classical carboxylic acid derivatives. In the last few years, the research on this topic has been flowered and remarkable progress has been made by different research groups. Specifically, vinyl and alkynyl substituted cyclic organic carbonates were used as allylic and propargyl surrogates upon decarboxylation toward the formation of various interesting *O*-heterocycles. In contrast, the corresponding vinyl and ethynyl cyclic organic carbamates also showed marvelous reactivities in the construction of a number of *N*-heterocycles.

In early 2018, Lu [5], Guo [6] and co-workers conducted a detailed review in this area. In the presence of transition metal catalysts, a number of structurally diverse N-heterocycles were synthesized using vinyl or ethynyl cyclic carbamates as starting materials [5]. Decarboxylation of cyclic carbonates proved to be a powerful tool to achieve chemo-, regio-, stereo- and enantioselective synthesis of complex structures [6]. As a related research topic, the Harrity group recently released a review of Pd-catalyzed cyclization reactions via *π*-allyl-Pd zwitterionic intermediates [7]. In order to avoid unnecessary duplication, we herein would like to focus on reviewing the latest contributions in the last two years, especially in transition metal-catalyzed decarboxylative transformations of cyclic carbonates and carbamates. We will first discuss the decarboxylative chemistry with cyclic carbamates as starting materials.

## 2. Transition Metal-Catalyzed Decarboxylation of Cyclic Carbamates

**Scheme 1:**
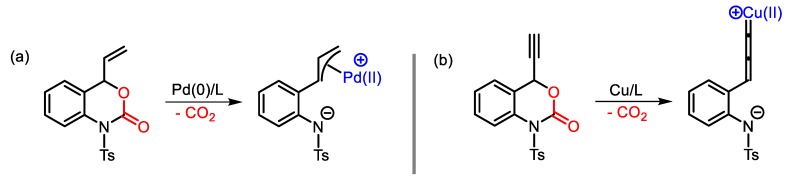
Palladium (**a**) or Copper (**b**) catalyzed decarboxylation of cyclic carbamates toward the formation of zwitterionic intermediates.

Cyclic carbamates have been widely used in the synthesis of a range of N-heterocyclic compounds through a key aza-quinone methide (aza-QM) intermediate. In the presence of a suitable palladium catalyst, the decarboxylation of vinyl cyclic carbamate would generate a zwitterionic intermediate (Scheme 1a) [8]. In contrast, the decarboxylation of ethynyl cyclic carbamate toward the formation of a zwitterionic intermediate generally requires an appropriate copper catalyst (Scheme 1b) [9]. In the presence of appropriate acceptors, a cyclization process would occur affording various interesting and useful N-heterocycles. In this section, the examples that have been reviewed by Lu and co-workers will not be discussed [5].

**Scheme 2:**
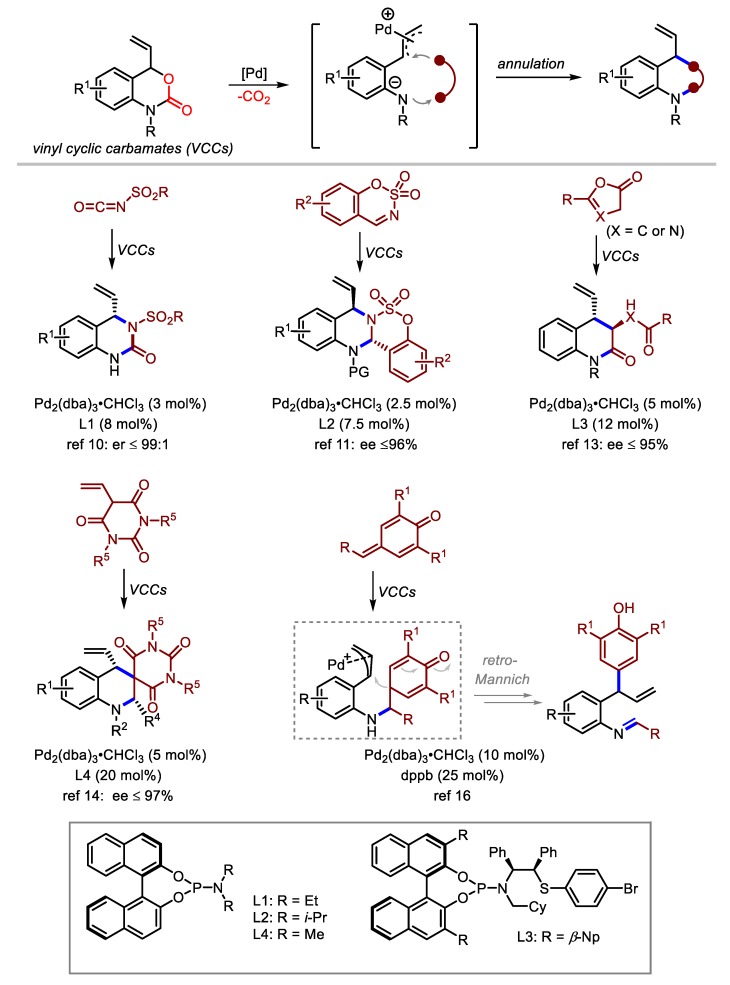
Pd-catalyzed decarboxylative cycloadditions of vinyl cyclic carbamates.

Decarboxylative cyclization of vinyl cyclic carbamates and benzylidene malononitriles toward the formation of chiral tetrahydroquinolines was first reported by Tunge et al. in 2008 [8]. In the following decade, cycloaddition reactions of vinyl cyclic carbamates with a variety of different electrophiles and nucleophiles have been boosted. In addition to previous achievements [5], new opportunities with neoteric acceptors have been discovered (Scheme 2). For instance, the decarboxylative formation of dihydroquinazolinones through a Pd-catalyzed [4 + 2] cycloaddition using sulfonyl isocyanates as electrophiles was reported by Shi and co-workers [10]. With the introduction of cyclic imines or barbiturate-based olefin reactants, it is feasible to construct highly functionalized quinazolines through decarboxylative cycloadditions [11,12,13]. Most recently, the annulation of deconjugated butenolides or azlactones with vinyl carbamates towards highly functionalized chiral dihydroquinol-2-ones was released by the Xiao group [14]. It was found that the utilization of the newly exploited chiral P,S-ligand and hydrogen bonding is the key to control the regioselectivity for this reaction. Apart from palladium catalysis, iridium/Brønsted acid co-catalytic system was applied for the preparation of quinolinones through a formal [4 + 2] cycloaddition by the Shi group [15]. Later, the same group developed a palladium-catalyzed cascade cyclization reaction of para-quinone methides and cyclic carbamates [16].

**Scheme 3:**
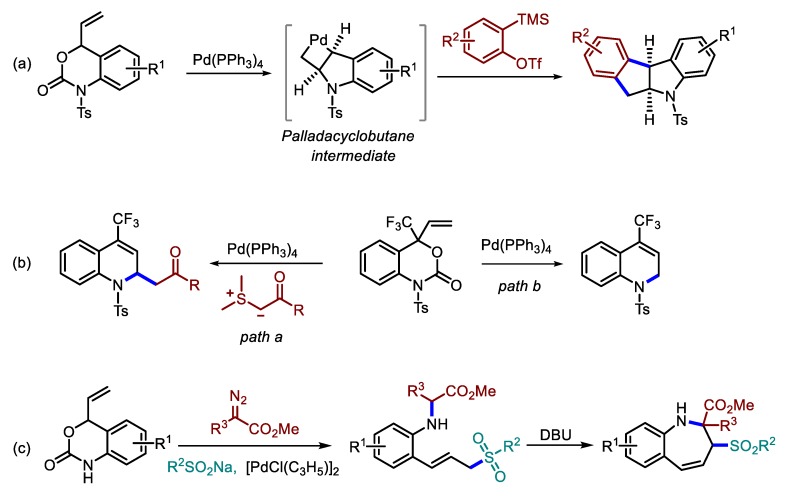
Palladium-catalyzed decarboxylative reaction of cyclic carbamates with different reaction partners: (**a**) with arynes; (**b**) with or without sulfur ylides and (**c**) three-component domino reactions.

Interestingly, the Zhai group developed a palladium-catalyzed decarboxylative coupling of arynes and vinyl benzoxazinanones toward the formation of functionalized indoles (Scheme 3a). In allylic chemistry, the nucleophilic attack of *π*-allyl palladium intermediate generally occurred at the terminal or internal carbon. However, this conversion features an intramolecular nucleophilic attack of the amide group at the central carbon of the *π*-allyl palladium intermediate, though a deeper understanding of the mechanism is still required [17]. The Shibata group reported an unprecedented intermolecular cyclization of cyclic carbamate and sulfur ylides toward the formation of 4-trifluoromethyl-dihyroquinolines (Scheme 3b, path a); without externally added acceptors, a cyclization process would also be possible through an intramolecular attack of the zwitterionic *π*-allyl intermediate (Scheme 3b, path b) [18]. In contrast, the same group also proved [4 + 1] diastereoselective intermolecular cyclization using sulfur ylides and non-vinyl-substituted benzoxazinanones, affording various trifluoromethyl-substituted indolines [19]. Moreover, a three-component domino reaction via decarboxylation, allylation, and N-H carbene insertion in the sequence was reported by Yang and co-workers toward the formation of various allylic sulfone-containing amino acid derivatives (Scheme 3c) [20].

**Scheme 4:**
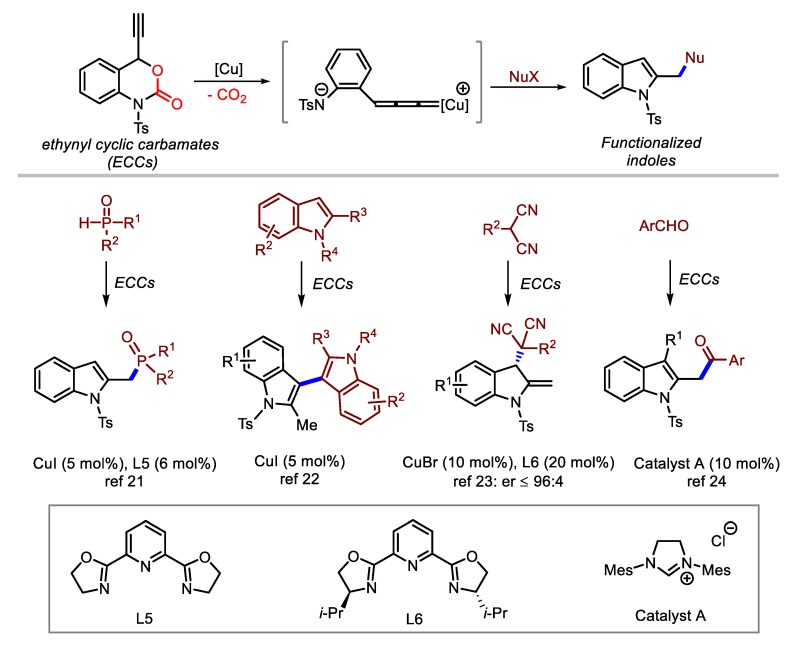
Decarboxylative conversions of ethynyl cyclic carbamates toward the formation of indole derivatives.

Comparatively, the decarboxylative annulation of ethynyl cyclic carbamates has mostly been achieved through Cu-allenylidene intermediate. The Cu-allenylidene dipole species could be trapped by different nucleophiles toward the formation of various functionalized indole skeletons (Scheme 4). In the presence of phosphonate nucleophile, 2-phosphorylmethyl indoles could be produced with this strategy [21]. The use of an indole nucleophile allows the facile synthesis of a variety of 3,3’-biindoles [22]. In the presence of an appropriate copper catalyst and chiral urea-cinchona organocatalyst, enantioselective decarboxylative propargylation and hydroamination of ethynyl benzoxazinanones were established affording chiral 3-indolin malononitrile derivatives [23]. Notably, the Zhao group reported a metal-free decarboxylative protocol utilizing propargylic carbamates and aldehydes as substrates. In this methodology, a range of 4-alkynyl dihydroquinolinones and 2, 3-difunctionalized indoles could be readily synthesized under the catalysis of *N*-heterocyclic carbene [24].

**Scheme 5:**
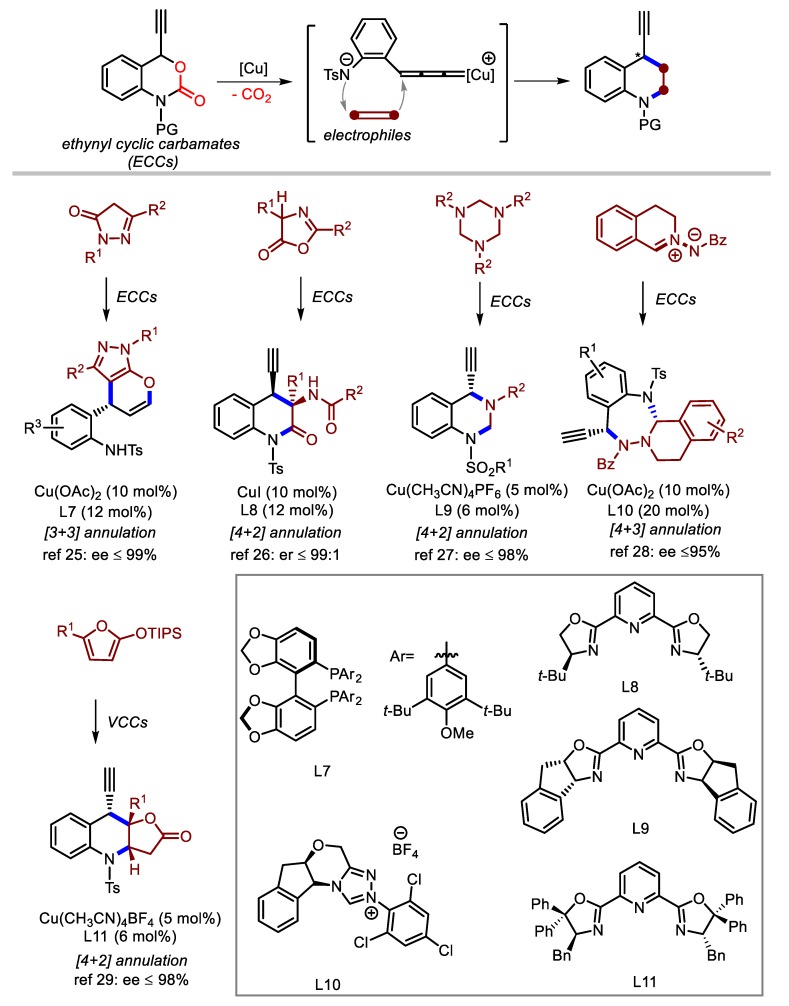
Cu-catalyzed decarboxylative annulation of ethynyl carbamates with different reagents.

In the presence of suitable reagents, the decarboxylative annulation process using ethynyl cyclic carbamates proved to be a feasible method for the preparation of *N*-heterocycles. In this sense, different chemicals such as pyrazolones, hexahydro-1,3,5-triazines, azlactones, and *C,N*-cyclic azomethineimines were submitted for the reactions toward the formation of various N-heterocycles (Scheme 5) [25,26,27,28]. It is worth noting that the Wu group accomplished an enantioselective [4 + 2] cycloaddition reaction of ethynyl benzoxazinanones and silyloxyfurans toward the formation of tetrahydroquinolines featuring three stereo carbon centers [29].

## 3. Transition Metal-Catalyzed Decarboxylation of Cyclic Carbonates

As stable and readily accessible allylic and propargylic donors, cyclic carbonates have emerged as highly reactive substrates in various stereo- and enantioselective reactions. In the past ten years, the synthetic potential of these cyclic carbonates has been greatly demonstrated in a wide variety of decarboxylative processes. Decarboxylation of vinyl cyclic carbonate with a palladium catalyst would generate a zwitterionic intermediate featuring a nucleophilic alkoxide and an electrophilic *π*-allyl-palladium site. This reactive zwitterionic species can serve as 1,3- or 1,5-dipole. The reaction of this dipole species with different cyclization acceptors would lead to formal [3 + 2], [5 + 2], [5 + 3] or [5 + 4] annulation reactions. 

**Scheme 6:**
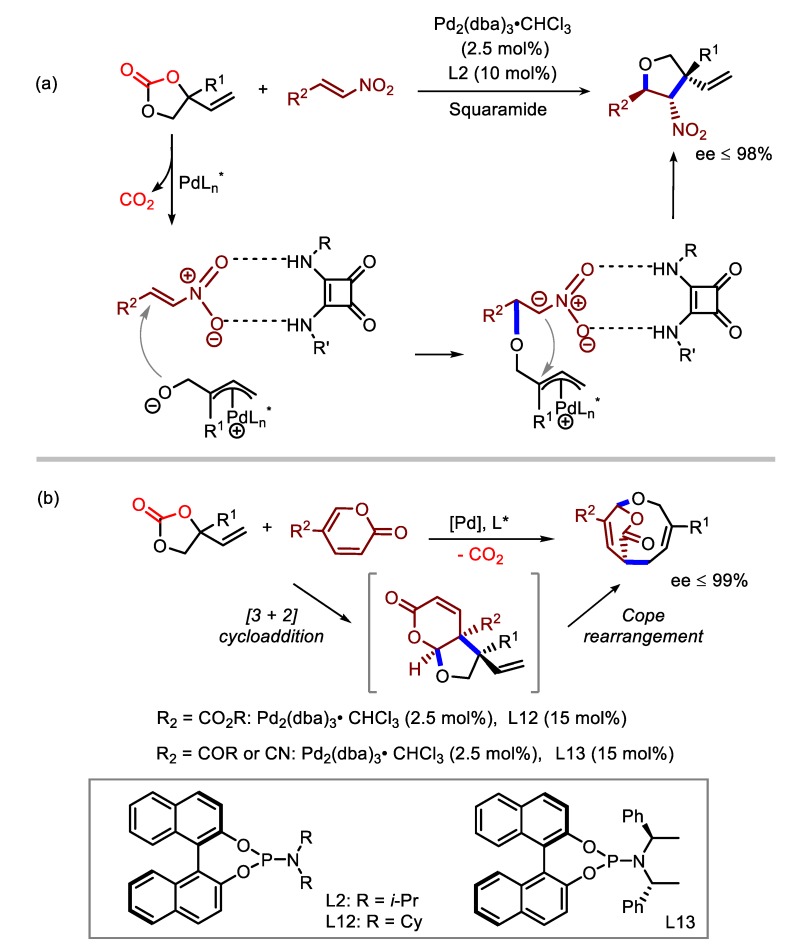
(**a**) Cooperative catalysis strategy for the cycloaddition of VECs with *β*-Nitroolefins and (**b**) [3 + 2] cycloaddition/Cope rearrangement reaction of VECs.

More recently, a palladium and squaramide co-catalyzed decarboxylative [3 + 2] cyclization of VCCs and *β*-nitroolefins was achieved by the Zhang group (Scheme 6a) [30]. The chiral squaramide proved to be vital for achieving high enantioselectivity. A similar strategy was utilized for the construction of furanbenzodihydropyran skeletons by the same group through a formal [3 + 2] cyclization with VCC as substrates [31]. With the introduction of coumalates as acceptors, the Guo group realized the chiral synthesis of nine-membered ethers with excellent enantioselectivities (mostly > 99% ee) through a tandem [3 + 2] cycloaddition followed by Cope rearrangement (Scheme 6b). The mechanism was further confirmed by DFT calculations [32].

**Scheme 7:**
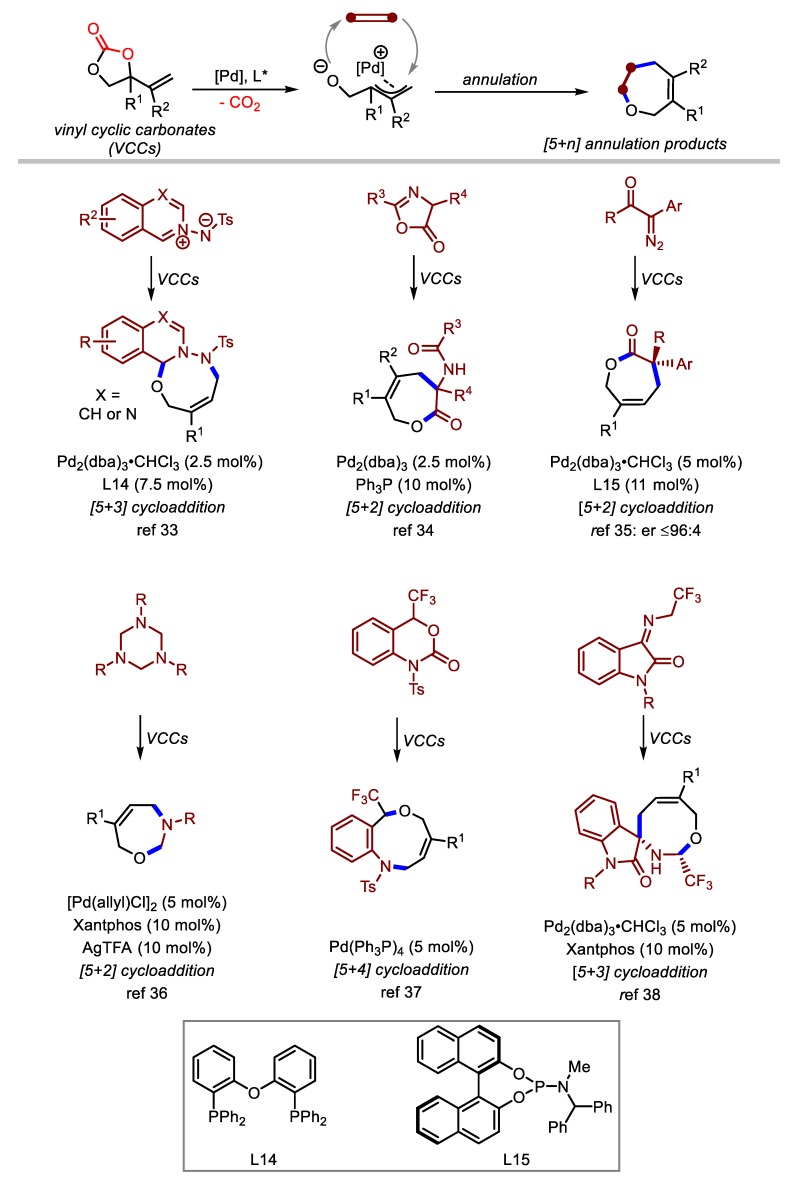
Decarboxylative [5 + n] cycloadditions of VCCs with various electrophiles.

In addition to these aforementioned [3 + 2] annulations, the decarboxylative formation of medium-membered heterocycles through a Pd-catalyzed [5 + n] annulation has been achieved by different research groups (Scheme 7) [33,34,35,36,37,38]. For example, the Xiao group realized an enantioselective [5 + 2] cycloaddition reaction of VCCs and *α*-diazoketones by merging photoactivation and Pd catalysis and a variety of seven-membered lactones bearing chiral quaternary stereocenters with high enantioselectivity were delivered through this methodology [35]. 

**Scheme 8:**
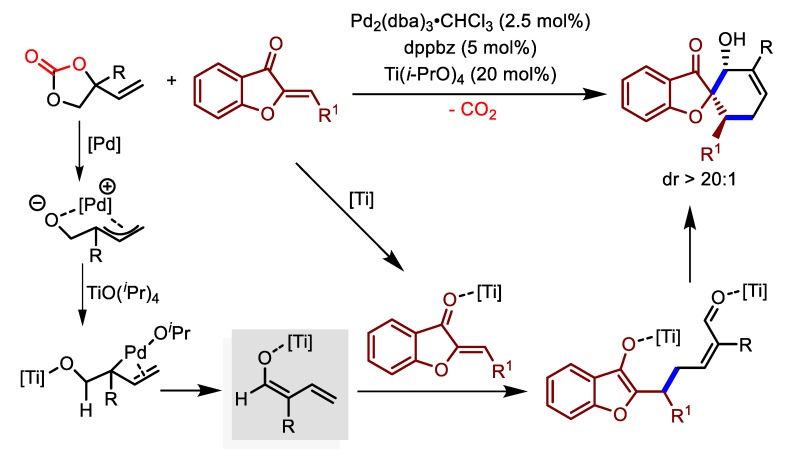
Palladium-titanium relay catalysis enabled umpolung reactivity in the synthesis of spiro-heterocycles.

Umpolung reactivity of the *π*-allyl zwitterionic species was first noted by Guo and co-workers [39] and later, it was further explored by the Zhao group [40]. In the presence of a palladium and titanium catalyst, the umpolung annulation process occurred with the use of aurones and VCC as starting materials toward the formation of [6,5] and [5,5] spiro-heterocycles bearing three contiguous stereocenters (Scheme 8). Mechanistically, it was proposed that the Pd-*π*-allyl intermediate first reacted with Ti(O*^i^*Pr)_4_, followed by ligand exchange between titanium and palladium resulting in a titanium-dienolate species through *β* elimination process. 

**Scheme 9:**
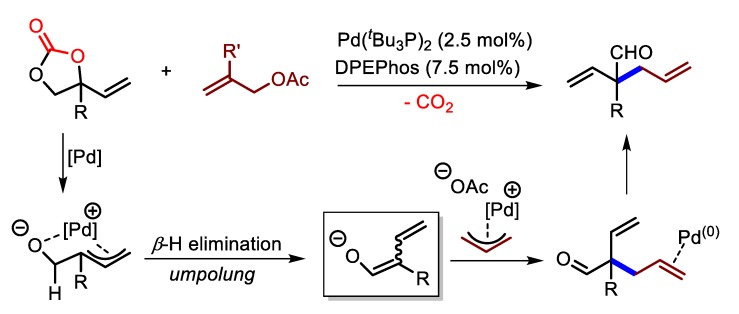
Pd-catalyzed decarboxylative transformations of VCCs toward highly functionalized aldehydes bearing quaternary carbon centers.

The Zhai group accomplished a Pd-catalyzed decarboxylative umpolung reaction of VCCs producing a variety of structurally diverse and synthetically useful all-carbon *α*-vinyl quaternary aldehydes (Scheme 9) [41]. The key step is that the *π*-allyl-Pd intermediate undergoes *β*-H elimination affording a nucleophilic dienolate. Most recently, a [3 + 3] cycloaddition of VCCs and triazinanes was achieved under the co-catalysis of palladium and Lewis acid towards the formation of polysubstituted tetrahydropyrimidines by the Yang group. Interestingly in this case, the vinyl carbonates served as a 3-carbon synthon, which is unique in this kind of decarboxylative cycloaddition process [42].

**Scheme 10:**
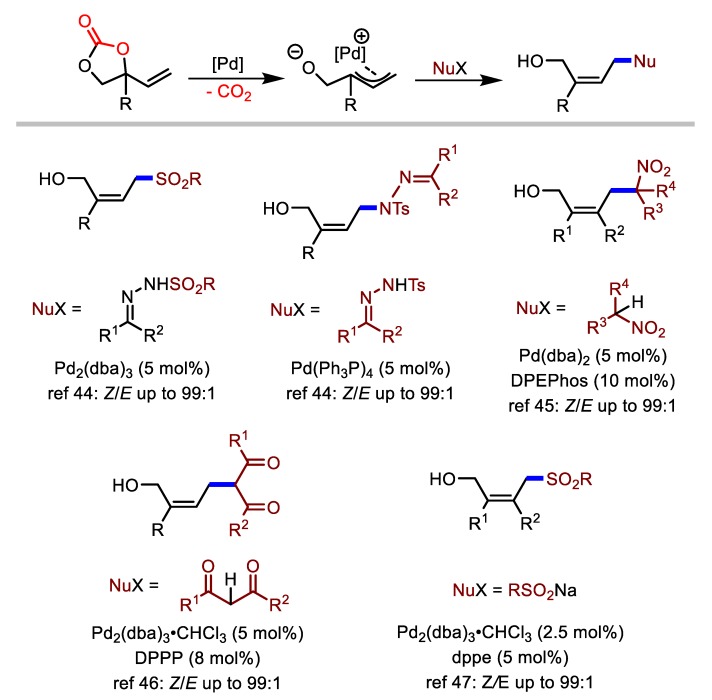
Pd-catalyzed decarboxylative formation of highly substituted (*Z*)-configured allylic alcohols from VCCs.

Unlike the cyclization process mentioned above, in the presence of suitable palladium catalyst and ligand, the stereoselective synthesis of a variety of highly functionalized allylic alcohols can be realized. This concept was first proved by Guo and co-workers using aryl amine nucleophiles toward the formation of highly functionalized (*Z*)-configured allylic alcohols/amines; the DFT (Density Functional Theory) studies suggested that the formation of a six-membered palladacyclic intermediate is the key for excellent stereocontrol [43]. Apart from amines, various other nucleophiles were demonstrated to be efficient for the syntheses of a huge number of allylic alcohols with excellent stereoselectivity (Scheme 10) [44,45,46,47]. By judicious choice of the palladium catalyst and ligand, the nucleophilic attack could be switched toward the sterically hindered carbon of the palladium allyl intermediate that derived from vinyl carbonate; this concept was first demonstrated by Guo and co-workers in the preparation of chiral *α,α*-disubstituted allylic aryl amines [48], and later it was further developed by the Khan group in the synthesis of chiral sulfones [49]. In the presence of naphthol nucleophile, the Liang group disclosed an unprecedented [3 + 2] or [3 + 3] cycloaddition reaction using VCC as substrates under palladium catalysis [50]. By simply switching the ligands, the cyclization mode can be readily controlled toward the formation of O-heterocycles. 

**Scheme 11:**
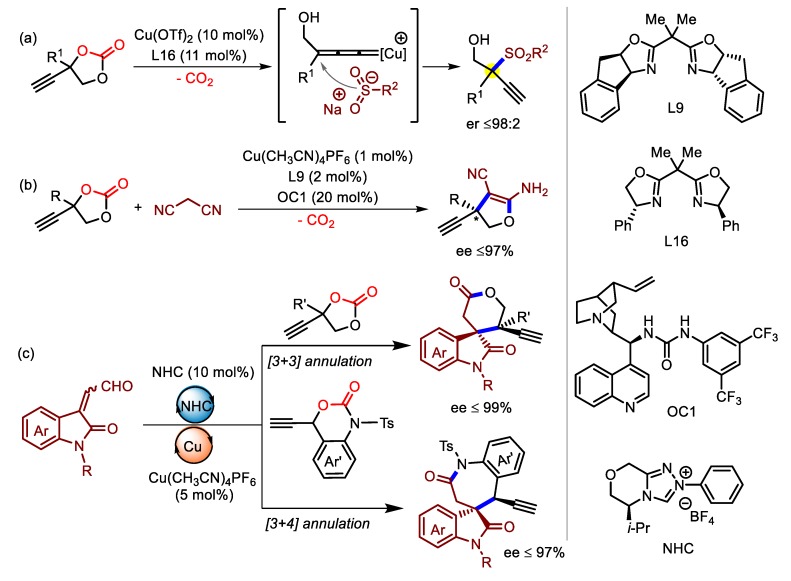
(**a**) Cu-catalyzed asymmetric propargylic sulfonylation; (**b**) asymmetric [3 + 2] reaction of carbamates with malononitrile and (**c**) [3 + 3] and [3 + 4] annulations of isatin-derived enals.

In contrast, the catalytic transformations of alkynyl-substituted cyclic carbonates were less investigated. The alkyne-functionalized carbonates were frequently used as important synthons in asymmetric propargylation reactions for the construction of quaternary stereocenters. In the presence of sodium sulfinate nucleophiles and copper catalyst, chiral propargylic sulfones can be synthesized with CO_2_ as the sole byproduct (Scheme 11a) [51]. Moreover, the coupling of malononitrile and alkynyl-carbonates proved to be feasible resulting in a series of chiral polysubstituted dihydrofurans with high enantioselectivities (up to 97% ee) (Scheme 11b) [52]. Most recently, the Gong group achieved the asymmetric synthesis of numerous spiro compounds with this strategy using both ethynylethylene carbonates and carbamates as starting materials through a NHC/copper cooperative catalytic system (Scheme 11c) [53].

## 4. Miscellaneous Substrates

**Scheme 12:**
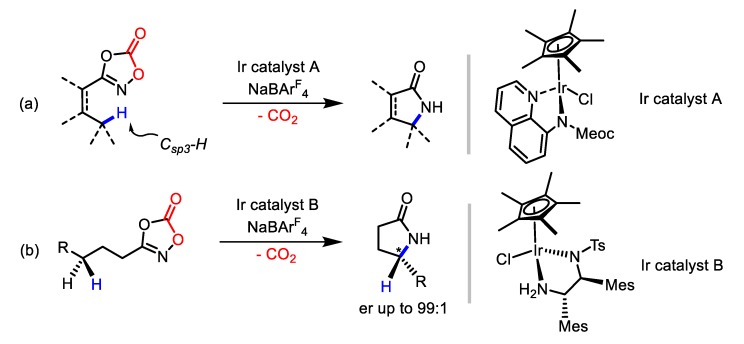
Selective formation of *γ*-lactams via C-H amidation.

1,4,2-Dioxazol-5-ones, known as dioxazolones, can be easily prepared from the corresponding commercially available alkyl carboxylic acids. These substrates are relatively easy to be activated under mild reaction conditions due to the presence of a weak N-O bond in the heterocycle. In general, due to the inherent instability, carbonyl nitrenes are prone to undergo Curtius rearrangement affording isocyanates as the main products. Based on the computational calculations reported by the Chang group [54,55], it was believed that the Curtius rearrangement is more sensitive to the charge variations of the metal center than the C-H insertion, thus electron-donating ligands may increase the Curtius-rearrangement barrier to a larger extent than the C-H insertion barrier. In early 2018, Chang and co-workers developed a method for the regioselective formation of *γ*-lactams with iridium catalysis (Scheme 12). The reactions proceeded smoothly via sp^3^ and sp^2^ C-H amidation with exceptional selectivity. The application potential of the methodology was further demonstrated by the late-stage functionalization of different amino acid derivatives and other bioactive compounds [54]. Enantioselective nitrene insertion to C(sp^3^)-H bonds was also developed by different research groups to afford chiral *γ*-lactams [55,56,57,58].

**Scheme 13:**
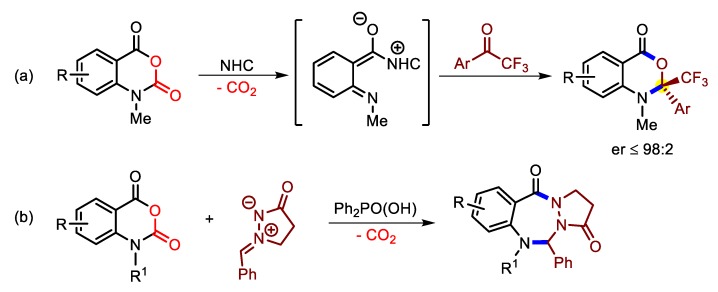
(**a**) NHC-catalyzed [4+2] cycloaddition of carbamates and CF_3_-containing partners and (**b**) brønsted acid-catalyzed [4 + 3] cyclization of carbamates with *N*,*N*’-cyclic azomethine imines.

A Rh-catalyzed three-component approach was reported for the synthesis of *α*-branched amines with terminal alkenes as substrates. The reactions could be carried out under mild conditions and tolerated well with different functionalities [59]. Regioselective amidation of allylic alkenes involving inert C-H activation with the use of 1,4,2-Dioxazol-5-ones as starting materials was also feasible utilizing Ir or Rh catalyst [60,61,62]. As analogs of cyclic carbamates, isatoic anhydrides have also emerged as powerful building blocks for the preparation of functionalized *N*-heterocycles. For example, Scheidt and co-workers disclosed an NHC-mediated [4 + 2] cycloaddition of isatoic anhydride and trifluoromethyl ketones. Various enantioenriched dihydrobenzoxazin-4-ones functionalized with a CF_3_ group can be produced with this protocol (Scheme 13a) [63]. In this transformation, the generation of an NHC-bonded intermediate is vital for the success of the reactions. Additionally, a Brønsted acid-catalyzed [4 + 3] cyclization of *N*,*N*’-cyclic azomethine imines with isatoic anhydrides was accomplished by the Shi group (Scheme 13b) [64].

## 5. Conclusions and Outlook

This review briefly summarizes the synthetic application of cyclic organic carbonates and carbamates with transition metal catalysis in the last two years. More and more novel catalytic transformations have been realized with efficient and vibrant reactivity and selectivity. The combination of transition metal catalysis with the use of cyclic carbamates as starting materials provides new strategies for the preparation of structurally diverse N-heterocycles. Cyclic carbonates played an important role in the synthesis of *O*-heterocycles. The merging of dioxazolones and transition metal catalysts proved to be a powerful tool to achieve challenging C–H bond functionalization toward the formation of interesting compounds. However, most of the decarboxylative transformations with cyclic carbamate and/or carbonate substrates require expensive transition metal catalysts. The development of interesting decarboxylative reactions with these cyclic structures as starting materials with earth-abundant metal alternatives as catalysts would be highly desired and of significance. Alternatively, the exploration of a metal-free strategy would be highly attractive for pharmaceutical purposes. Furthermore, the combination of transition metal catalysts with photochemistry, radical chemistry or organocatalyst may bring new opportunities during the exploration of novel and interesting chemistry.
